# Highly Thermoconductive, Strong Graphene-Based Composite Films by Eliminating Nanosheets Wrinkles

**DOI:** 10.1007/s40820-023-01252-w

**Published:** 2023-11-17

**Authors:** Guang Xiao, Hao Li, Zhizhou Yu, Haoting Niu, Yagang Yao

**Affiliations:** 1 College of Engineering and Applied Sciences, Jiangsu Key Laboratory of Artificial Functional Materials, and Collaborative Innovation Center of Advanced Microstructures, National Laboratory of Solid State Microstructures, Nanjing University, Nanjing, 210093 People’s Republic of China; 2 Institute of Laser Manufacturing, Henan Academy of Sciences, Zhengzhou, 450052 People’s Republic of China; 3 Center for Quantum Transport and Thermal Energy Science, School of Physics and Technology, Nanjing Normal University, Nanjing, 210023 People’s Republic of China

**Keywords:** Graphene, Aramid nanofiber, Wrinkles elimination, In-plane stretching, Thermal conductivity

## Abstract

Graphene-based thermally conductive composites have been proposed as effective thermal management materials for cooling high-power electronic devices. However, when flexible graphene nanosheets are assembled into macroscopic thermally conductive composites, capillary forces induce shrinkage of graphene nanosheets to form wrinkles during solution-based spontaneous drying, which greatly reduces the thermal conductivity of the composites. Herein, graphene nanosheets/aramid nanofiber (GNS/ANF) composite films with high thermal conductivity were prepared by in-plane stretching of GNS/ANF composite hydrogel networks with hydrogen bonds and *π*–*π* interactions. The in-plane mechanical stretching eliminates graphene nanosheets wrinkles by suppressing inward shrinkage due to capillary forces during drying and achieves a high in-plane orientation of graphene nanosheets, thereby creating a fast in-plane heat transfer channel. The composite films (GNS/ANF-60 wt%) with eliminated graphene nanosheets wrinkles showed a significant increase in thermal conductivity (146 W m^−1^ K^−1^) and tensile strength (207 MPa). The combination of these excellent properties enables the GNS/ANF composite films to be effectively used for cooling flexible LED chips and smartphones, showing promising applications in the thermal management of high-power electronic devices.

**Supplementary Information:**

The online version contains supplementary material available at 10.1007/s40820-023-01252-w.

## Highlights

- Highly thermally conductive composite films with elimination of graphene nanosheet wrinkles prepared by a constrained drying strategy of in-plane stretching.
- Reveals the mechanism of graphene nanosheet wrinkle elimination to improve the thermal conductivity of composite films and demonstrates its use as a heat sink film for cooling flexible LED chips and smartphones.

**Supplementary Information:**

The online version contains supplementary material available at 10.1007/s40820-023-01252-w.

## Graphical abstract

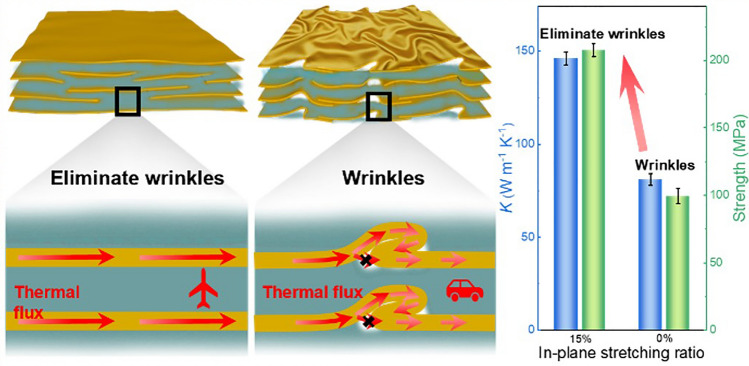

## Introduction

The continuing miniaturization and high integration of microelectronic components has brought the issue of heat dissipation into sharper focus [1, 2]. For example, high-density integrated circuit boards, chips, LED, and other electronic components generate a significant amount of heat during operation, creating an urgent need for materials with high thermal conductivity to solve their overheating problems, thereby reducing equipment temperatures and improving the performance and longevity of electronic devices [3–8]. Polymer-based thermally conductive composites, which are composed of thermally conductive fillers and polymers, show promise in realizing the synergistic advantages of the two components, resulting in high thermal conductivity and excellent mechanical properties composite materials [9–14]. They address to the thermal management needs of emerging technologies such as flexible electronic devices and represent a highly promising class of thermal management materials [15–19].

Graphene displays an ultra-high in-plane thermal conductivity, due to its perfect two-dimensional structure [20, 21]. Its unique two-dimensional morphology and high aspect ratio make it an ideal thermal filler for fabricating thermally conductive composite materials with multilayer nanostructures, thereby promoting accurate and rapid in-plane heat transfer [22–24]. Currently, many efforts have been made to improve the thermal conductivity of graphene-based thermal composites [25–28]. Typically, a high content of graphene filler is added to a polymer and then the interfacial interaction between the thermal filler and the polymer matrix is enhanced by surface modification (such as fluorination [29, 30], oxidation [31] or addition of surface modifiers [32]) to improve the dispersion and prevent graphene agglomeration. However, these approaches tend to compromise the lattice integrity of graphene, resulting in a reduction in its intrinsic thermal conductivity [33, 34]. A more effective approach is to improve the in-plane orientation of the two-dimensional thermal filler in the composite material to construct an in-plane oriented thermal network, thereby enabling rapid phonon transport [35–37]. Various methods have been explored to prepare graphene–polymer mixed solutions into solid thermal composites with layered in-plane orientation structures, including vacuum-assisted filtration [38–40], evaporation-induced self-assembly [32], layer-by-layer assembly [41] and sol–gel-film conversion approach [42–44]. Nevertheless, it should be noted that the solvent removal step is a critical operation in the above solution assembly techniques [45, 46]. During the drying process, solvent evaporation generates capillary forces that cause the large-sized graphene nanosheets with flexibility to shrink inward, eventually forming wrinkles and introducing air bubbles inside the composite [47–51]. Wrinkled graphene nanosheets reduce the density of the composite material, increase the air interface and increase phonon scattering, thereby reducing the thermal conductivity of the composite material [52–55].

**Fig. 1:**
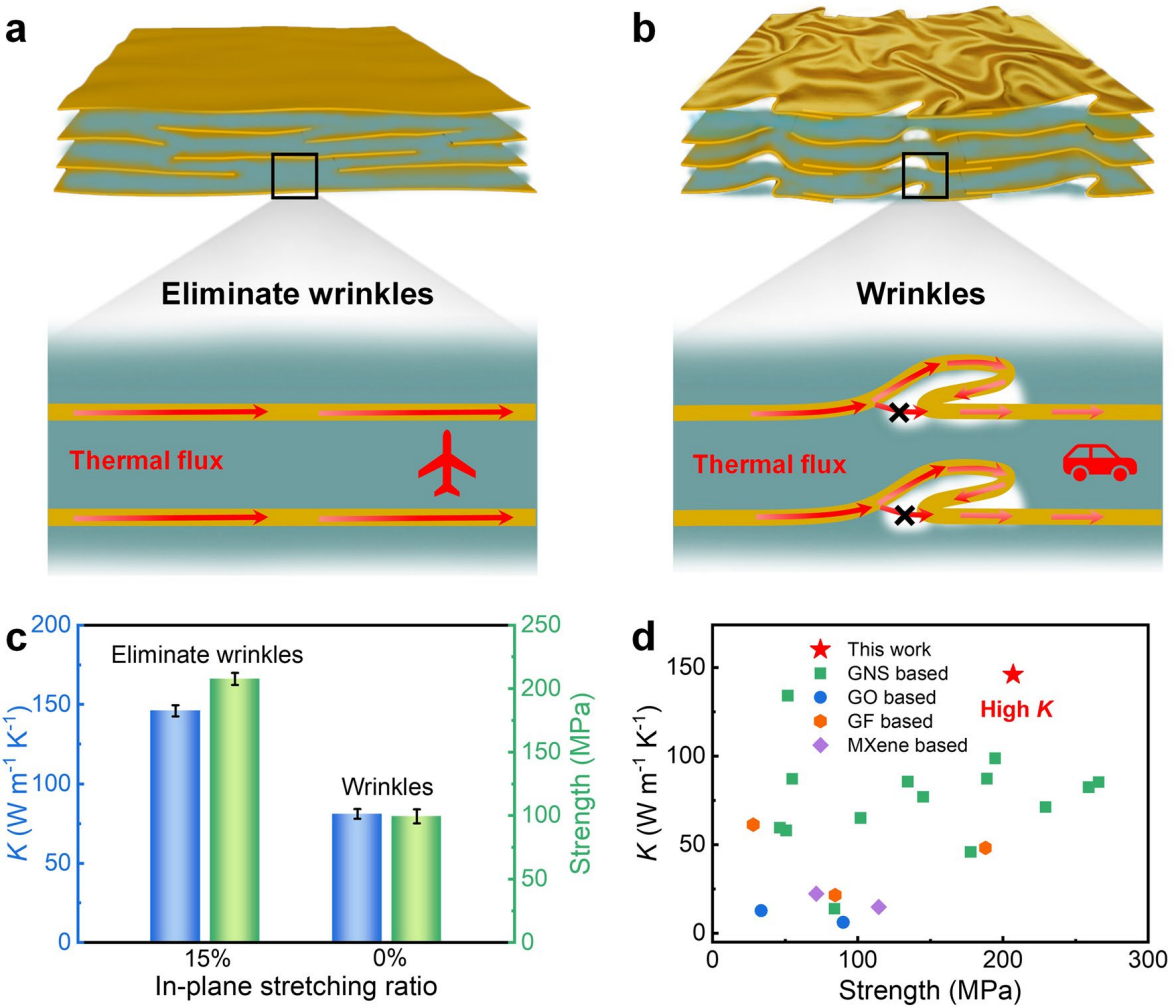
Thermal conductivity and mechanical properties of GNS/ANF composite films with eliminated nanosheets wrinkles. **a, b** Schematic diagram of heat transfer in composite films with eliminated nanosheet wrinkles and with nanosheet wrinkles. **c** Comparison of thermal conductivity and tensile strength of the composite films with eliminated nanosheets wrinkles and with nanosheet wrinkles. **d** Comparison of thermal conductivity and tensile strength of the composite films with eliminated nanosheets wrinkles with previously reported thermal management materials with nanosheets wrinkles. Reference data are listed in Table S1

Previous methods of composite assembly seldom provide controlled constraints during drying, so it is difficult to achieve wrinkle removal of 2D graphene nanosheet fillers in composites, thus limiting further improvement of their thermal conductivity. In this study, we integrated the sol–gel film transformation method with the in-plane stretching-constrained assembly method. Robust GNS/ANF composite hydrogels were first prepared, followed by in-plane stretching of the composite hydrogels and maintaining the constrained state until drying was completed. This process eliminated wrinkles in the graphene nanosheets and forced them to align along the in-plane, which facilitates the fast phonon transfer in the horizontal plane (Fig. 1a). Compared to the composite films with graphene nanosheet wrinkles (Fig. 1b), the thermal conductivity and tensile strength of the composite films with graphene nanosheet wrinkles eliminated were significantly improved. Specifically, the thermal conductivity increased from 81 to 146 W m^−1^ K^−1^, and the tensile strength increased from 99 to 207 MPa (Fig. 1c), which also outperformed various previous thermally conductive composites with nanosheets wrinkles, including graphene-based [32, 38–41, 56–59], graphene oxide-based [31, 60], fluorinated graphene-based [29, 30], and MXene-based [61, 62] composites (Fig. 1d).

The significant improvement in thermal conductivity can be attributed to the following factors: (i) During in-plane stretching, the mechanical tension not only counteracts the contraction effect of the capillary force, but also spreads and flattens the flexible large-size graphene nanosheets along the in-plane direction. This process eliminates the wrinkles of graphene nanosheets, as well as the air bubbles introduced by the wrinkles, making the structure of GNS/ANF composite films more compact. The oriented and ordered stacking of GNS is favorable for the rapid phonon transfer along the plane. (ii) There is a strong* π*–*π* conjugation effect between the ANF molecular chain and GNS. The wrinkles of graphene nanosheets are opened, exposing more area for contact with ANF molecular chains. The GNS/air interface is effectively reduced, and the interfacial interaction between GNS and ANF is enhanced, which effectively reduces the interfacial thermal resistance. With such high thermal conductivity and excellent mechanical properties, constrained dried GNS/ANF composite films exhibit excellent heat dissipation performance in cooling high-power electronic devices, such as flexible LED chips and smartphones, and have broad application prospects in fields such as electronics and 5G technology. At the same time, we believe that eliminating nanosheets wrinkles by in-plane stretching strategy provides a feasible path for other composite materials to improve their thermal conductivity.

## Experimental Section

### Materials

High-quality graphene nanosheets (GNS, 5–15 μm in diameter, average thickness 1.8 nm) were purchased from Deyang Carbenene Technology Co., Ltd. AMF (Kevlar 29) was purchased from Dongguan SOVETL Co., Ltd. Potassium tert-butoxide was provided from Sigma-Aldrich, and dimethyl sulfoxide (99.7%, Aladdin) was used as received.

### Preparation of Constrained Dried GNS/ANF Films

**Fig. 2:**
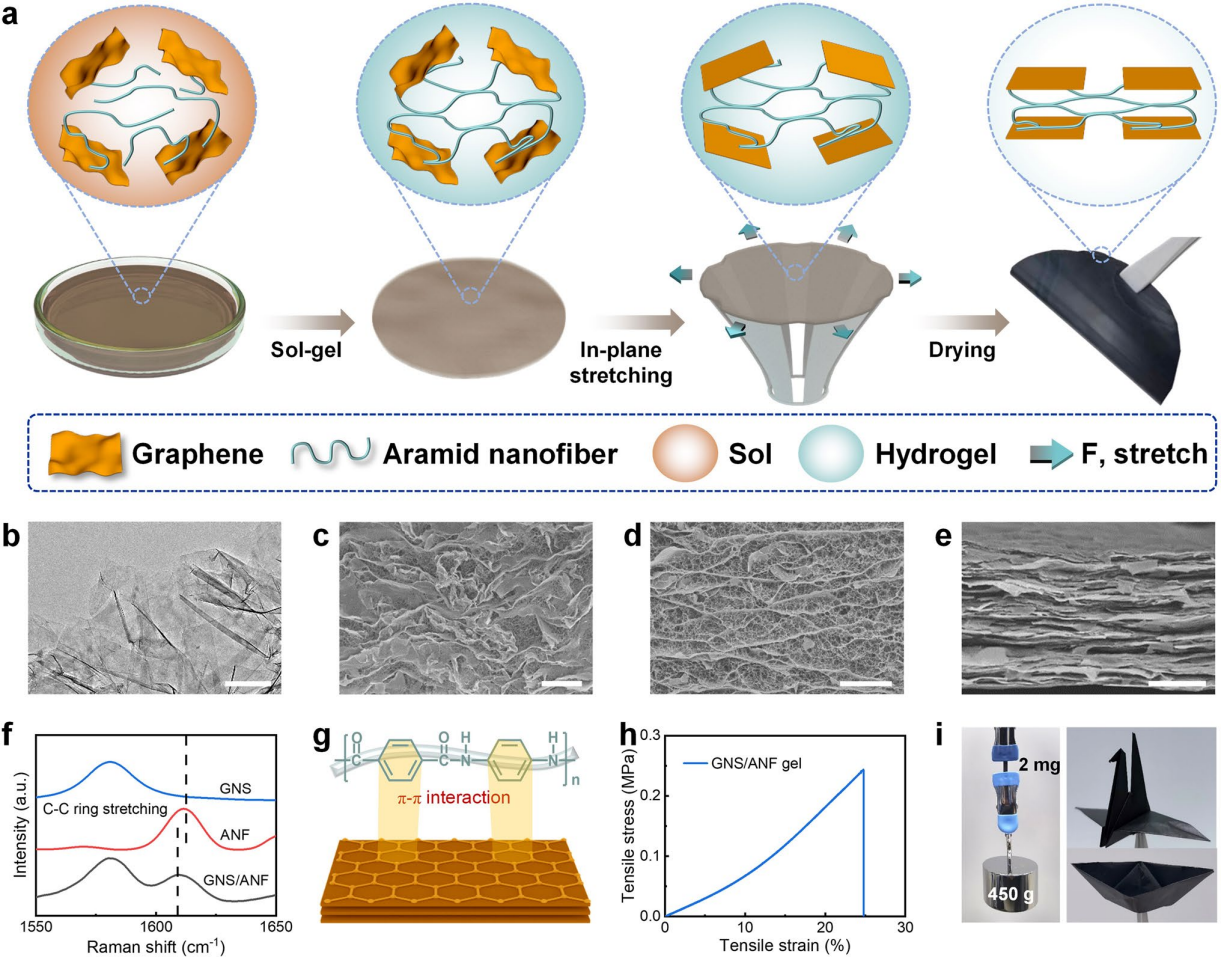
Preparation of GNS/ANF films with eliminated nanosheets wrinkles by in-plane stretching strategy. **a** Schematic illustration of the fabrication process of GNS/ANF films with eliminated nanosheets wrinkles. **b** TEM image of graphene with wrinkles. **c** SEM image of spontaneously dried GNS/ANF composite aerogel. **d, e** SEM image of constrained dried GNS/ANF composite aerogel and films. **f, g** Raman spectra of GNS, ANF, GNS/ANF composite films and illustration of the existence of *π*–*π* interaction between GNS and ANF. **h** Stress–strain curves of GNS/ANF composite hydrogels.** i** GNS/ANF composite films with eliminated nanosheets wrinkles can lift heavy weights and be folded into origami crane and boat. Scale bar: 500 nm (**b**), 10 μm (**c**, **d**), 3 μm (**e**)

Constrained dried GNS/ANF films were prepared by in-plane stretching of GNS/ANF composite hydrogels. Six grams of AMF and 4.5 g potassium tert-butanol were added to 189.5 g dimethyl sulfoxide, and the mixture was mechanically stirred for 12 h to obtain dark red, viscous ANF solution (3 wt%). GNS (0.75 g) was added to dimethyl sulfoxide solution (32.58 g) and dispersed by sonication for 2 h (800 W, 40 kHz). Then, 3 wt% ANF solution (16.67 g) was added and mechanical stirring was continued for 12 h to form uniformly dispersed GNS/ANF-60 wt% sol. The GNS/ANF sol was poured into a Petri dish and then immersed in water to form a black GNS/ANF hydrogel, which was rinsed with repeatedly using deionized water to completely remove dimethyl sulfoxide and potassium tert-butanol. The mass fraction of GNS in the composite hydrogel was 1.5 wt% and the mass fraction of ANF was 1 wt%. The GNS/ANF hydrogels were stretched in-plane with a stretching device and kept under stretching constraint until drying, as shown in Fig. 2a. The stretching ratio in diameter is controlled to be 0, 5, 10 and 15%. For example, GNS/ANF composite films with 60 wt% graphene content with a 15% in-plane stretch ratio is named GNS/ANF-15%.

### Characterization

**Fig. 3:**
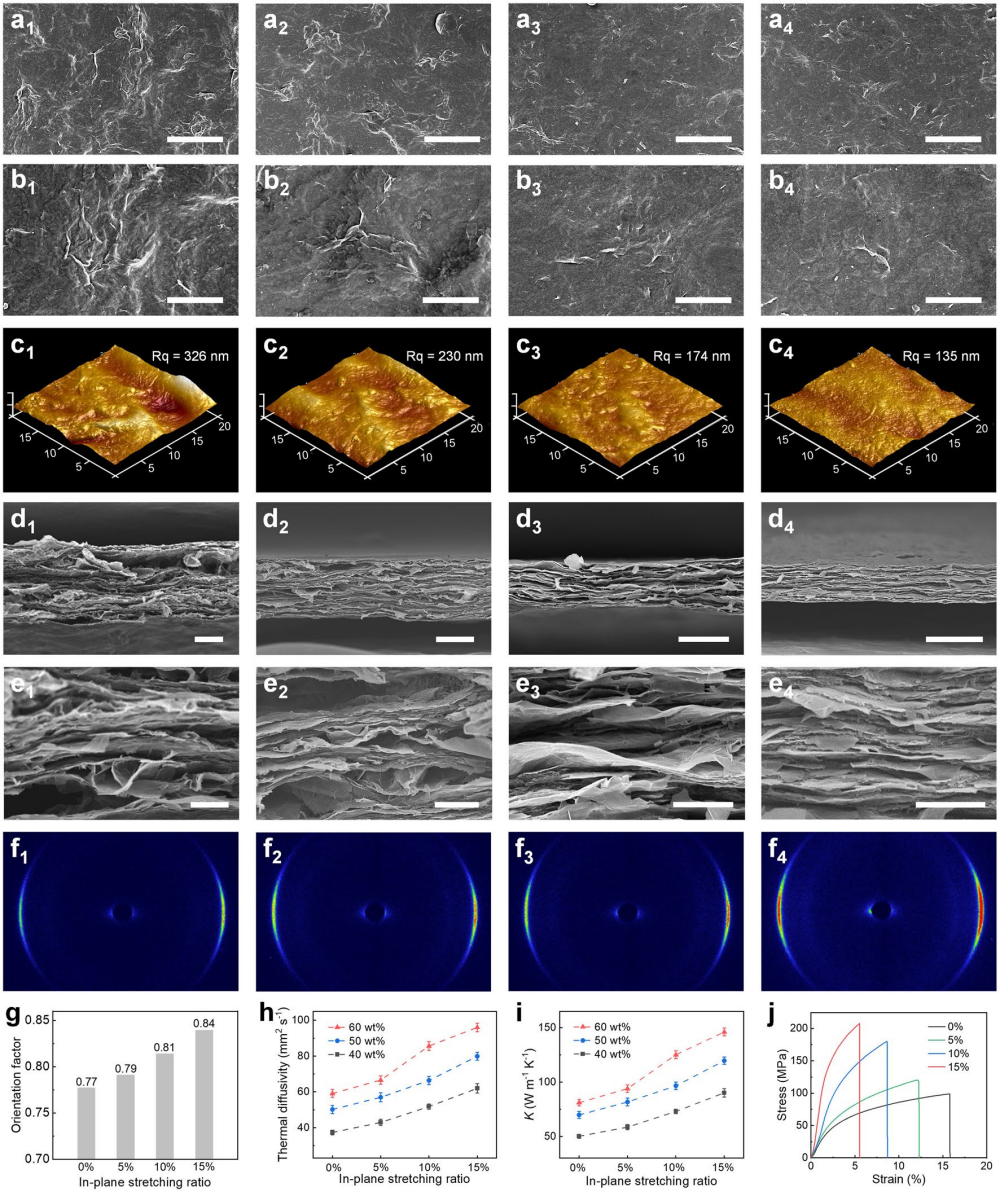
Wrinkles regulation and thermal conductivity enhancement of composite films. **a, b** Surface SEM images. **c** AFM images. **d, e** Cross-sectional SEM images and **f** WAXS patterns for GNS/ANF films by in-plane stretching for (1) 0%; (2) 5%; (3) 10%; and (4) 15%. **g** Orientation factor change. **h, i** Thermal diffusivity and thermal conductivity of GNS/ANF-60, 50, 40 wt% films by different in-plane stretching ratio. **j** Tensile stress–strain curves of GNS/ANF-60 wt% films by different in-plane stretching ratio. Scale bar: **a** 30 μm, **b** 10 μm, **d** 10 μm, **e** 3 μm

SEM images were obtained with a field-emission scanning electron microscope (S-8100, Hitachi). TEM images were obtained with a transmission electron microscope (Tecnai G2 F20, FEI). AFM images were obtained with an atomic force microscope (Dimension Icon, Bruker). The test area of Fig. 3c is 400 μm^2^. Raman spectra were recorded by microscopic confocal Raman spectrometer (ARAMIS HORIBA) with an excitation wavelength of 532 nm. XPS data were obtained using an X-ray photoelectron spectrometer (K-Alpha, Thermo Fisher Scientific). FTIR spectra were collected using a Nicolet IS50 FTIR spectrometer (Thermo Fisher Scientific). X-ray diffraction (XRD) patterns were obtained using a LabX XRD-6000 (SHIMADZU) instrument with Cu Kα radiation at a scanning speed of 10° min^−1^. Tensile properties were measured on a Shimadzu AGS-X Tester. The thermal conductivity of the nanocomposite was calculated with $K = \alpha \times \rho \times C_{p}$, where *K*,* α*, $\rho$ , and *C*_p_ represent thermal conductivity, thermal diffusivity, density, and heat capacity, respectively. Thermal diffusivity was measured using the laser flash method (LFA 447, NETZSCH). Density was obtained by the calculation of sample volume and weight. The specific heat capacity was obtained by differential scanning calorimetry (Q2000, TA). Infrared thermal images were taken with an infrared thermal imager (FOTRIC 345). The electrical conductivity was analyzed by four-probe method (ST2258C, Suzhou Jingge Electronic Co., Ltd.). Thermogravimetric analysis (TGA) was performed with a simultaneous thermal analyzer (STA 449 C, NETZSCH) at a heating rate of 10 °C min^−1^ under air atmosphere.

## Results and Discussion

### Preparation of Composite Films with Eliminated Graphene Nanosheet Wrinkles

GNS/ANF composite films with eliminated graphene nanosheets wrinkles were prepared using an in-plane stretching strategy. As shown in Fig. 2a, based on the sol–gel-film transformation approach, a mixed sol of aramid nanofibers and graphene nanosheets was re-protonated in water to assemble a GNS/ANF composite hydrogel with a three-dimensional network framework structure of nanofibers. The GNS/ANF composite hydrogel was then stretched outward along the plane using an in-plane stretching device and kept in a restrained state until drying was completed. Large-sized graphene nanosheets with an average thickness of 1.8 nm and a diameter of 5–15 μm, which have very few lattice defects (*I*_D_/*I*_G_ = 0.07, *C*/*O* = 47.1, Fig. S1) were utilized in this study. It is worth noting that graphene nanosheets have numerous edge folds and wrinkles, which will make it difficult to achieve an orderly alignment of graphene nanosheets in composites (Fig. 2b). The ANF were re-protonated and entangled to form a three-dimensional nanofiber network (average diameter 15.3 nm, Fig. S2). The GNS/ANF composite hydrogel was subjected to freeze-drying to obtain the GNS/ANF composite aerogel, which was then employed to observe the state changes of GNS. SEM images revealed that in the normal spontaneously dried composite aerogel, the GNS exhibited a plethora of wrinkles and is disordered in orientation (Fig. 2c), while in the constrained dried composite aerogel, the wrinkles of graphene nanosheets are eliminated and aligned along the horizontal force direction (Figs. 2d and S3). Cross-sectional SEM images of the dried GNS/ANF composite films indicated that the wrinkling of the graphene nanosheets was eliminated, resulting in a highly oriented stacking and compact structure (Fig. 2e). EDS images confirmed the uniform distribution of carbon, oxygen, and nitrogen elements in the cross section of the GNS/ANF composite films (Fig. S4).

The aramid nanofiber network in the GNS/ANF composite hydrogel has strong hydrogen bonding interactions. Meanwhile, the Raman spectroscopic results reveal that compared with pure ANF, the peak representing the C − C ring stretching of aramid molecules at 1610 cm^−1^ in the GNS/ANF composite films is left-shifted, indicating a charge transfer between graphene and the large aromatic molecule, confirming a strong *π*–*π* interaction between graphene and aramid nanofiber (Fig. 2f, g) [63–65]. These interfacial interactions result in excellent tensile properties of the GNS/ANF composite hydrogel, with a maximum breaking strength of 0.24 MPa and a maximum breaking strain of 24.7% (Fig. 2h), making it suitable for subsequent in-plane tensile constrained assembly. The GNS/ANF composite films with eliminated nanosheets wrinkles exhibit outstanding mechanical performance, capable of lifting weights up to 225,000 times its own weight, and can be folded into origami crane and boat (Fig. 2i).

### Wrinkles Regulation and Thermal Conductivity Enhancement of Composite Films

The in-plane stretching was further investigated to control the structure of the constrained GNS/ANF composite films, and the morphology of the GNS/ANF composite films with different in-plane stretching ratios was characterized. As the in-plane stretching ratio gradually increased from 0 to 15%, the SEM images show that the folding streaks on the surface of the composite films significantly decreased and became smoother and flatter, indicating that the folded graphene nanosheets was stretched and flattened under the stretching constraint (Fig. 3a, b). The AFM images show a gradual reduction in the height of the raised surface folds of the composite films from 1286 to 318 nm, corresponding to a gradual reduction in the surface roughness from 326 to 135 nm (Fig. 3c). The cross-sectional SEM images of the composite films show that the orientation of graphene nanosheets gradually increases with the increase of the in-plane stretching ratio. The high magnification SEM images show that the previously wrinkled graphene nanosheets is stretched and flattened along the in-plane by mechanical restraint, accompanied by the extrusion of air bubbles from the wrinkles. The in-plane oriented GNS and ANF are alternately stacked to form a dense and orderly nanofiber layered framework structure (Fig. 3d, e).

In-plane stretching enhances the orientation alignment of graphene nanosheets, which is verified by 2D-WAXS [66]. X-ray beams were irradiated into the interior of the films from the side. The results show that the equatorial streak in the scattering pattern gradually becomes stronger as the in-plane stretching ratio increases (Fig. 3f), and the orientation factor also increases from 0.77 to 0.84 (Fig. 3g). The in-plane stretching forces the composite films into a more oriented and compact structure, giving the composite films enhanced thermal and mechanical properties. As the in-plane stretching ratio increases from 0 to 15%, the thermal diffusivity of GNS/ANF-60 wt% gradually increases from 59.1 ± 2.2 to 96.0 ± 2.3 mm^2^ s^−1^ (Fig. 3h) and the thermal conductivity increases from 81.0 ± 3.0 to 146.0 ± 3.5 W m^−1^ K^−1^ (Fig. 3i and Table S2). The stress–strain curve shows that the tensile strength of GNS/ANF-60 wt% also increases from 99 MPa at 0% in-plane stretch to 207 MPa at 15% in-plane stretch (Figs. 3j and S5), while the Young's modulus also increases monotonically to 8.2 ± 0.5 GPa at 15% in-plane stretch (Fig. S6 and Table S3). The composite films with graphene nanosheets folds eliminated by the in-plane stretching assembly strategy also exceed the values reported for previous work with various spontaneous assembly methods in terms of thermal conductivity and tensile strength (Fig. S7 and Table S4).

The crack propagation and fracture surface morphology of the composite films were further characterized. The 15% in-plane stretching ratio of the composite films was flat and smooth around the cracks. The GNS was oriented in the horizontal force direction at the crack fracture and was firmly attached to the ANF. However, the composite films with 0% in-plane stretching ratio are rough and wrinkled, and the graphene sheets are loose and disordered at the crack fracture (Fig. S8). These results demonstrate that the in-plane stretching effectively improves the stacking orientation of graphene nanosheets and increases the tensile strength of the composite films. Interestingly, the electrical conductivity of the composite films gradually increased with the increase of the in-plane stretching ratio, indicating that graphene nanosheets with wrinkles eliminated by in-plane stretching build better conductive paths (Fig. S9).

### Principles of Enhancing the Thermal Conductivity of Composite Films

**Fig. 4:**
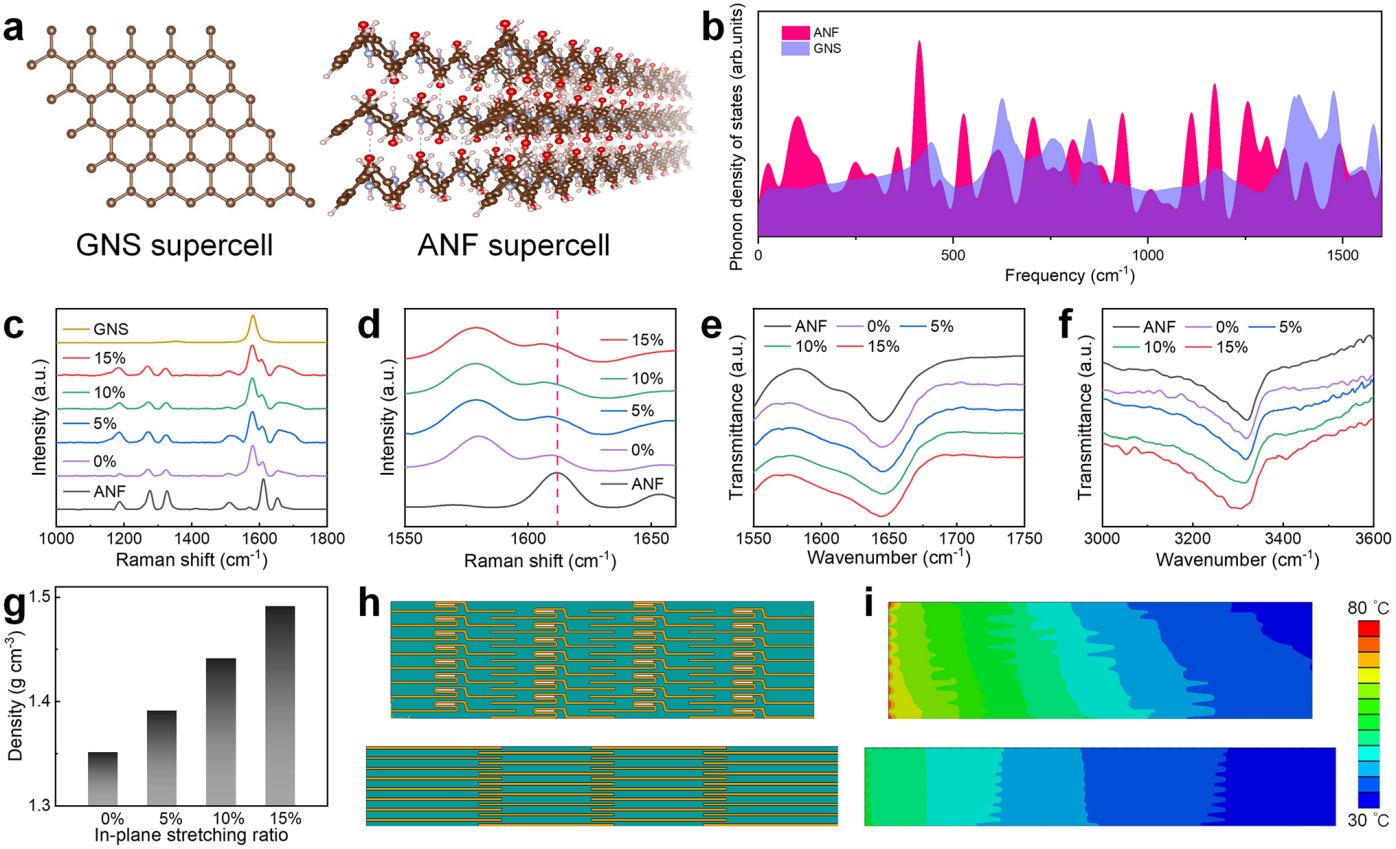
Principle of improving the thermal conductivity of GNS/ANF films by eliminating GNS wrinkles through in-plane stretching. **a** Schematic diagram of the GNS supercell, ANF supercell. **b** The PDOS versus frequency results of GNS and ANF. **c, d** Raman spectra of ANF films, GNS and GNS/ANF films with different in-plane stretching ratio. **e, f** FTIR spectra. **g** Density for GNS/ANF films by different in-plane stretching ratio. **h, i** Modeling and temperature distribution of GNS/ANF films by in-plane stretching for 0% (up), and 15% (down)

To investigate the mechanism of in-plane stretching constrained dried on the enhancement of thermal conductivity of GNS/ANF composite films, models were constructed for GNS and ANF (Fig. 4a). The phonon density of states (PDOS) was subsequently calculated, and the resulting PDOS versus frequency is shown in Fig. 4b [67]. The significant overlap between GNS and ANF suggests a good phonon spectral match, indicating that effective energy transfer can occur at the GNS-ANF interface, thereby facilitating highly efficient phonon collision. In addition to the matched phonon vibration modes, the strength of the interfacial interaction also determines the efficient phonon transfer. The composite films with different in-plane stretching ratios were characterized by Raman spectroscopy (Fig. 4c, d). The results show that as the stretching ratio increases from 0 to 15%, the peak at 1610 cm^−1^ is increasingly shifted to the left toward 1605 cm^−1^, indicating that the *π*–*π* interaction between GNS and ANF becomes stronger. To further confirm this strengthening of the *π*–*π* interaction, we examined the XPS spectra of C 1 s. As the in-plane stretching ratio increased, the peak representing the *π*–*π* interaction gradually shifted from 290 to 290.8 eV, consistent with the Raman spectroscopy results (Fig. S10) [68]. Meanwhile, the FTIR spectroscopy results show that the peaks near 1645 cm^−1^ (C=O stretching vibration) and 3326 cm^−1^ (N–H stretching vibration) were observed to broaden and shift to the left (Fig. 4e, f), indicating that the hydrogen bonding of the ANF network within the composite films was stronger after in-plane stretching [68, 69].

After in-plane stretching, the GNS is stretched out to eliminate the folds, resulting in a tighter bond with ANF, resulting in stronger interfacial interactions. In addition, the GNS is expanded and tiled along the in-plane direction to eliminate air bubbles contained within the wrinkles. The alternate stacking of GNS and ANF in an orderly orientation forms a more compact and orderly structure, and the density of the composite films gradually increases (Fig. 4g). All these results greatly improve the thermal conductivity of the composite films. Finite element simulations were used to illustrate the thermoconductive superiority of in GNS/ANF films that eliminate GNS wrinkles (Fig. 4h). Based on the transient thermal response, the temperature distribution along the in-plane direction of the sample was simulated by placing the heat source on the left side of the sample. Compared to the composite films with GNS wrinkles, the composite films with eliminated GNS wrinkles exhibit lower temperature on the right side because its ultra-high in-plane thermal conductivity allows the fastest in-plane heat transfer (Fig. 4i). It is worth noting that in the real world, thermal management materials rely on fast in-plane heat transfer to rapidly reduce the temperature of hot spots, which means that the in-plane thermal conductivity of the material plays a key role in effective heat dissipation. In summary, composite films with eliminated graphene nanosheets wrinkles exhibit better thermal performance and can dissipate accumulated heat faster.

### Thermal Management Demonstration of Composite Films

**Fig. 5:**
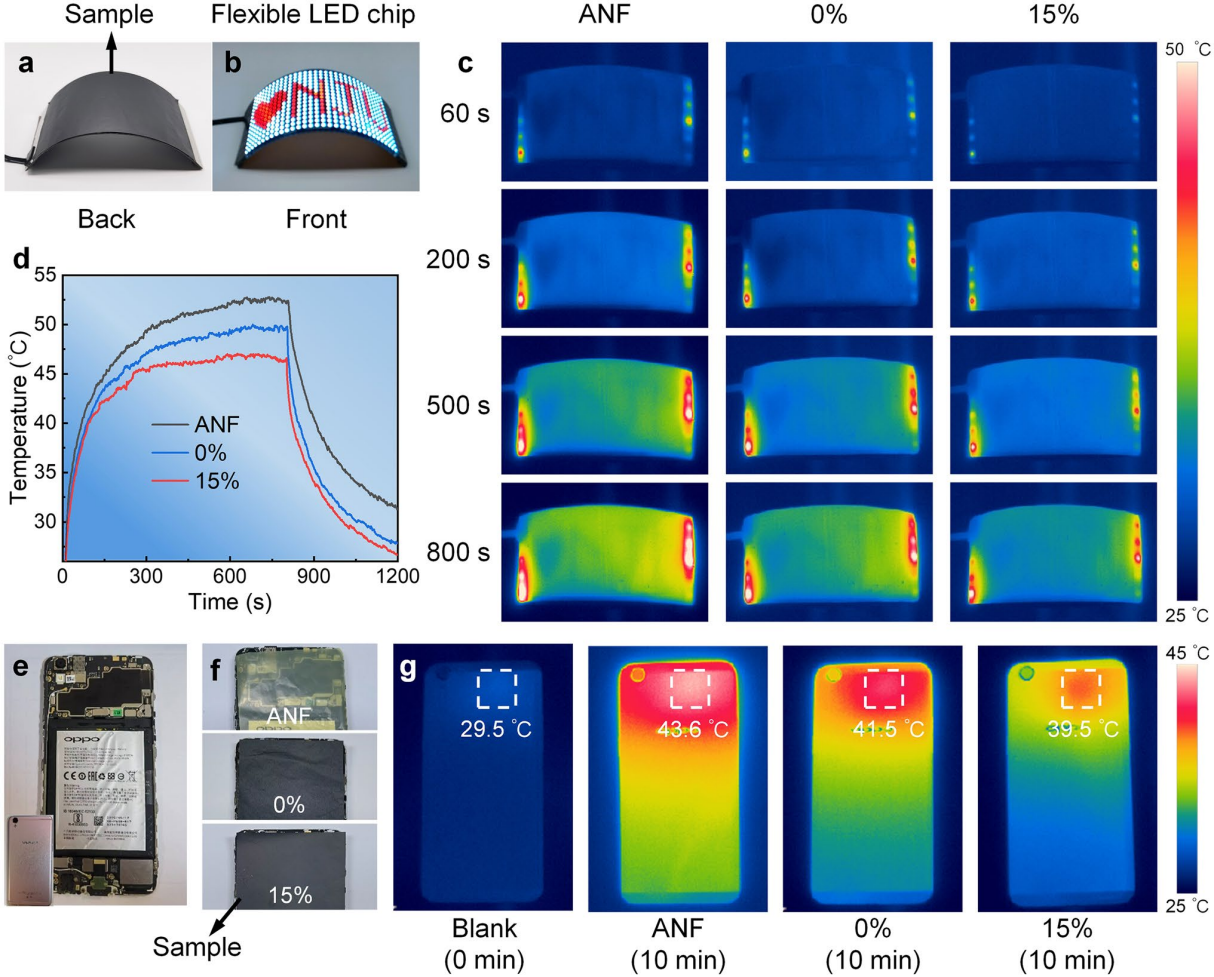
Thermal management demonstration of GNS/ANF composite films with eliminated GNS wrinkles. **a** GNS/ANF composite films integrated into the backside of a flexible LED chip. **b** Image of the front side. **c** Infrared thermal images. **d** Surface temperature evolution curves of a flexible LED chip with working time. **e, f** Pure ANF, GNS/ANF-0% and GNS/ANF-15% integrated into a smartphone. **g** Thermal infrared images of smartphone under different working conditions

The excellent thermal conductivity and mechanical properties of the GNS/ANF composite films with eliminated GNS wrinkles have been demonstrated, and its application as a thermal management material for cooling flexible LED chips and smartphones will now be discussed. As shown in Fig. 5a, the composite films sample is attached to the backside of the flexible LED chip, which was then turned over, with the front side facing up and kept in a bent state (Fig. 5b). The power was then turned on, and the temperature of the flexible LED chip surface was recorded using an infrared camera. The infrared thermal images show that the surface temperature of the flexible LED chip with integrated composite films with eliminated GNS wrinkles (GNS/ANF-15%) decreases to different degrees between 0 and 800 s compared with the pure ANF films and the composite films with GNS wrinkles (GNS/ANF-0%) (Fig. 5c). Their temperature evolution curves with working time are shown in Fig. 5d. At 800 s, the maximum surface temperature of the flexible LED chip with integrated GNS/ANF-15% is 46.6 °C, which is significantly lower than that of the pure ANF at 52.5 °C and GNS/ANF-0% at 49.8 °C. When the power is disconnected, the flexible LED chip with integrated GNS/ANF-15% has a sharp temperature drop during cooling, and the final cooling temperature is 26.8 °C, so its cooling efficiency is much higher than the above two.

In addition, these films were also integrated into the internal back of the smartphone (Fig. 5e, f), and their surface temperature evolution curves with working time were recorded by infrared thermal imager (Fig. S11). After the smartphone ran a large game program for 10 min, the maximum temperature on the backside of the phone supported by the pure ANF films and GNS/ANF-0% increased from 29.5 to 43.6 and 41.5 °C, respectively (Fig. 5g). In contrast, the smartphone supported by GNS/ANF-15% with excellent thermal conductivity exhibited a maximum temperature of 39.5 °C, which was 4.1 and 2.1 °C lower than the two above. Additionally, the overall temperature on the back of the smartphone was lower and more evenly distributed, indicating that GNS/ANF-15% with high in-plane thermal conductivity could quickly dissipate the intense heat generated by the smartphone chip. Furthermore, the composite films also exhibit extremely high thermal stability, with TGA tests showing that the composite films only begin to degrade significantly at 513 °C (Fig. S12).

## Conclusions

In summary, ultra-high in-plane thermal conductivity (146 W m^−1^ K^−1^), high tensile strength (207 MPa) GNS/ANF-60 wt% composite films were prepared based on the in-plane stretching strategy and sol–gel-film transformation approach. The remarkable improvement in thermal conductivity is attributed to the in-plane stretching strategy, which effectively eliminates the graphene nanosheets wrinkles, improves the orientation of GNS along the in-plane direction, and further enhances the interfacial interaction between GNS and ANF. We believe that the GNS/ANF composite films with such exceptional thermal conductivity and mechanical properties hold promising applications for various thermal management applications in the electronics and automotive industries.

## Supplementary Information

Below is the link to the electronic supplementary material.

Supplementary file1 (PDF 1352 KB)
