# Supplementary material for: Highly Thermoconductive, Strong Graphene-Based Composite Films by Eliminating Nanosheets Wrinkles
Source: Nanomicro Lett. 2023 Nov 17;16:17. doi: 10.1007/s40820-023-01252-w (PMC10656391; doi:10.1007/s40820-023-01252-w)
Supplement: Supplementary file 1 — Supplementary file1 (PDF 1352 KB) [file 40820_2023_1252_MOESM1_ESM.pdf]

Supporting Information for

Highly Thermoconductive, Strong Graphene-Based Composite Films by Eliminating Nanosheets Wrinkles

Guang Xiao[1, #], Hao Li[2, #], Zhizhou Yu[3], Haoting Niu[1] and Yagang Yao[1, *]

[1] National Laboratory of Solid State Microstructures, College of Engineering and Applied Sciences, Jiangsu Key Laboratory of Artificial Functional Materials, and Collaborative Innovation Center of Advanced Microstructures, Nanjing University, Nanjing 210093, P. R. China

[2] Institute of Laser Manufacturing, Henan Academy of Sciences, Zhengzhou, 450052, P. R. China

[3] Center for Quantum Transport and Thermal Energy Science, School of Physics and Technology, Nanjing Normal University, Nanjing 210023, P. R. China

[#]Guang Xiao and Hao Li contributed equally to this work.

*Corresponding author. E-mail: ygyao2018@nju.edu.cn (Yagang Yao)

S1 Supporting Methods

S1.1 Calculation of Herman's Order Parameter

Wide-angle X-ray scattering (WAXS) measurement was conducted on a Xenocs Xeuss 2.0 System by using an incident Cu−Kα X-ray beam that is parallel to film plane. X-ray wavelength, spot size, and distance between specimen and detector were 0.1542 nm, 172 × 172 μm^2, and 88 mm, respectively. Samples were square slices with a side length of 10 mm. Scattering patterns were collected by a Pilatus 300 k detector. Orientation factor (f) was calculated to describe orientation degree of GNS. Its value ranges from 0 to 1, where the former corresponds to an isotropic structure and the latter corresponds to a perfect orientation structure along the director. A Maier−Saupe distribution function was used to fit the azimuthal profile.

$$I = I_0 + A\exp[\omega\cos^2(\varphi - \varphi_0)]$$

where I_0 is the free baseline, φ is the azimuthal angle, φ_0 is the azimuth at the position of maximal intensity, and ω is a parameter that determines the width of the distribution. After the fitting, parameter ω was obtained, and the orientation factor f can be determined using the following formula

$$f = \frac{\int_{-1}^{1} P_2(\cos\varphi)\exp(\omega\cos^2\varphi)\ d(\cos\varphi)}{\int_{-1}^{1}\exp(\omega\cos^2\varphi)\ d(\cos\varphi)}$$

where the function $P_2(\cos\varphi)$ is the second-order Legendre polynomial of cos φ and often referred to as the Herman's order parameter function:

$$P_2(\cos\varphi) = \frac{1}{2}(3\cos^2\varphi - 1)$$

S1.2 Phonon Density of States (PDOS) Analysis

The first-principles calculations are performed using the Vienna Ab initio Simulation Package (VASP) based on the density functional theory (DFT). The generalized gradient approximation (GGA) of the Perdew-Burke-Ernzerhof (PBE) form is used for the exchange-correlation functional. The second-order harmonic interatomic force constants (IFCs) are calculated through the Hessian matrix using the density functional perturbation theory (DFPT). The phonon frequencies are then obtained using the PHONOPY package. To calculate the IFCs, a 4×4×1 supercell with a 3×3×1 q-point mesh is employed for graphene, and a 1×1×2 supercell with a 1×1×3 q-point mesh is employed for ANF.

For graphene, the kinetic energy cutoff is set to 500 eV and a 12 × 12 × 1 Monkhorst-Pack k-point mesh is adopted. The structure is fully relaxed until the force on each atom is less than 10^{-4} eV/Å. The calculated lattice constant of graphene is 2.46 Å. For the aramid nanofiber (ANF), the lattice constants of the primitive cell for the p-phenylene terephthalamide (PPTA) are set to a=15.74 Å, b=15.54 Å, and c=12.9 Å. Then the one-dimensional ANF is constructed by a 2×3×1 supercell and the chain orientation is the z-axis. A vacuum space of 15 Å is used to avoid interaction between two adjacent supercells. The kinetic energy cutoff is set to 450 eV and a 1 × 1 × 3 Γ-centered k-point mesh is adopted. The structure is fully relaxed until the force convergence threshold of 0.02 eV/Å is satisfied.

S1.3 Finite Element Mode

The heat transfer processes of spontaneously dried and constrained dried GNS/ANF-60 wt% films are modeled in COMSOL multiphysics software, which used a transient simulation model. Herein, the in-plane and out-of-plane thermal conductivity of GNS/ANF-0 % films are set to be 81.0 W m^{-1} K^{-1} and 0.41 W m^{-1} K^{-1}. The in-plane and out-of-plane thermal conductivity of GNS/ANF-15% films are set to be 146.0 W m^{-1} K^{-1} and 0.79 W m^{-1} K^{-1}. The ambient temperature is 25 °C.

S2 Supplementary Figures

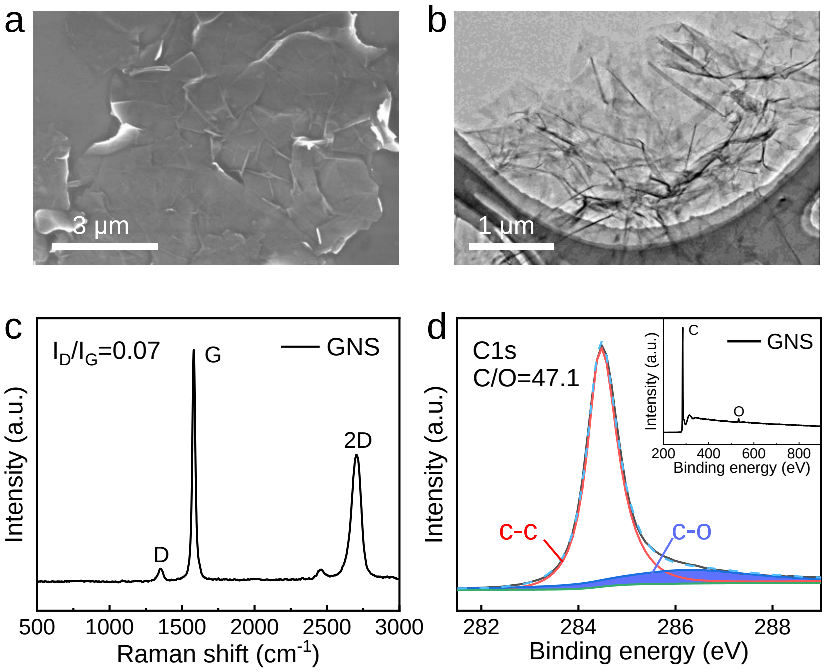

Fig. S1 Characterization of graphene nanosheets. **a** SEM and **b** TEM images of graphene nanosheets. **c** Raman spectra and **d** XPS spectra of graphene nanosheets

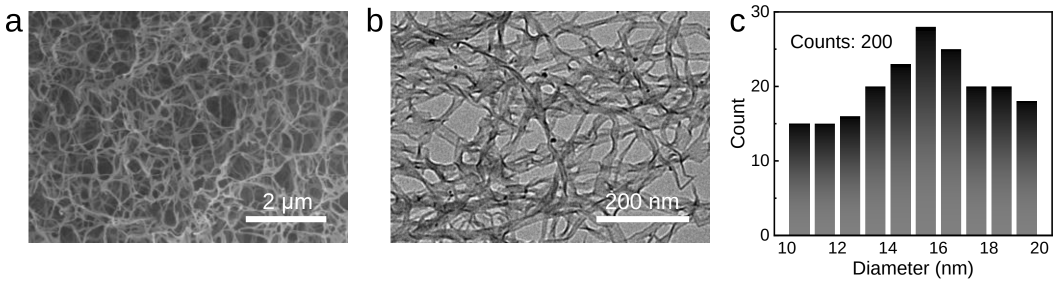

Fig. S2 Characterization of aramid nanofiber. **a** SEM and **b** TEM images of ANF. **c** Statistical data showing the diameter of ANF

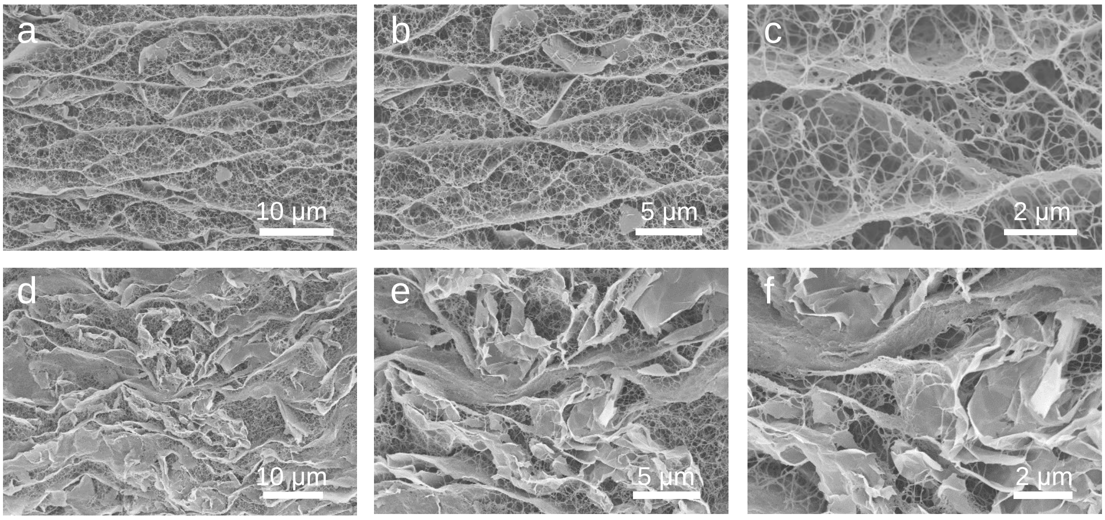

Fig. S3 SEM images for GNS/ANF composite aerogel with different in-plane stretching ratio. **a-c** 15%, **d-f** 0%

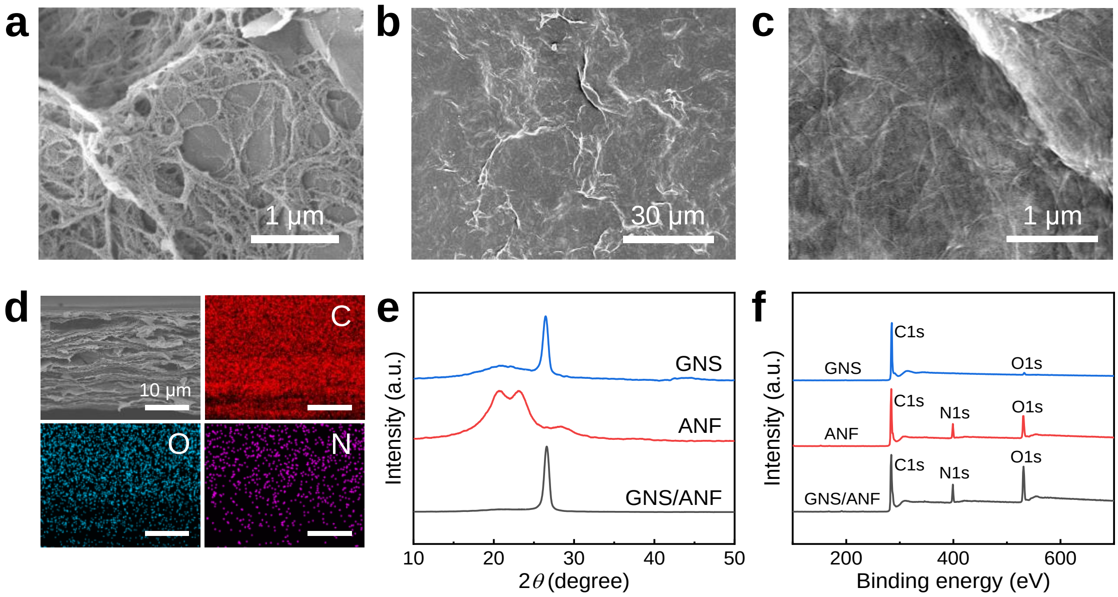

Fig. S4 Characterization of GNS/ANF composite films. **a** SEM image of aerogel. **b, c** Surface SEM image. **d** Cross-sectional SEM images along with elemental mapping images. **e** XRD patterns. **f** XPS spectra

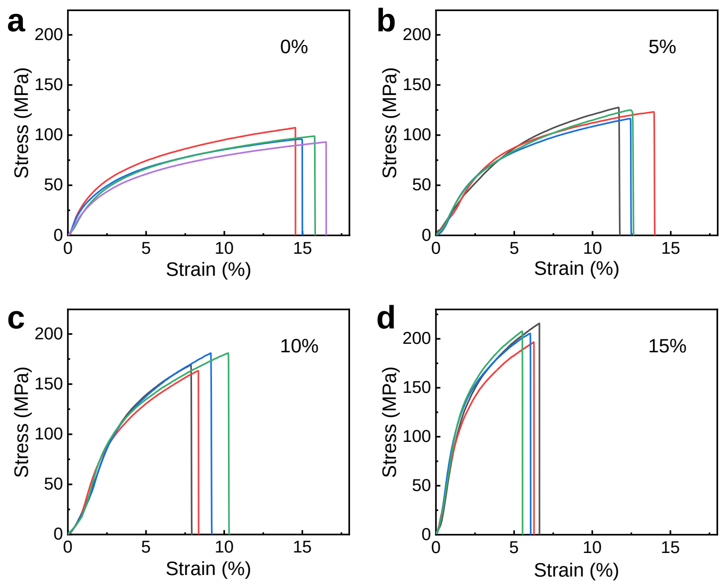

Fig. S5 Tensile stress-strain curves of GNS/ANF-60 wt% films by different in-plane stretching ratio. **a** 0%, **b** 5%, **c** 10%, and **d** 15%

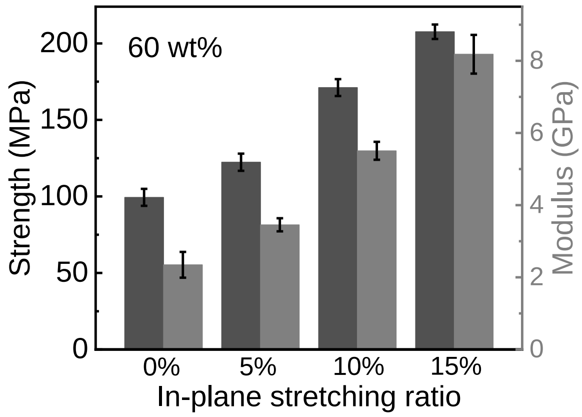

Fig. S6 Strength and modulus of GNS/ANF-60 wt% films by different in-plane stretching ratio

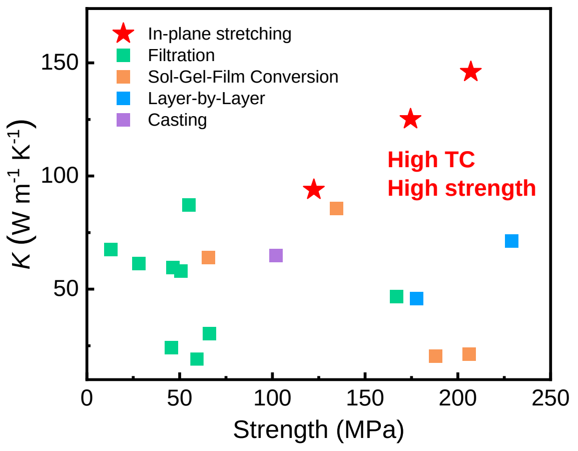

Fig. S7 Comparison of thermal conductivity and tensile strength of composite films assembled by in-plane stretching and previously reported spontaneous assembly thermal management materials

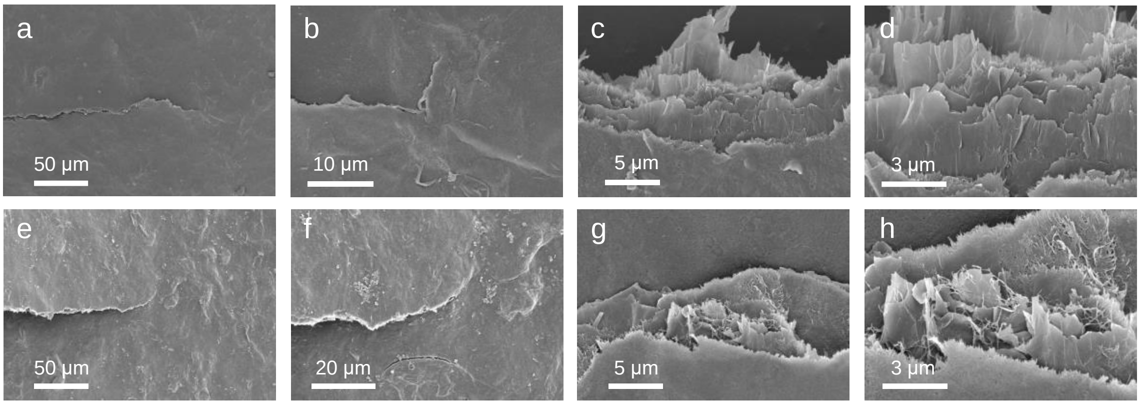

Fig. S8 Fracture behavior of GNS/ANF-60 wt% films by different in-plane stretching ratio. **a-d** 15%, **e-h** 0%

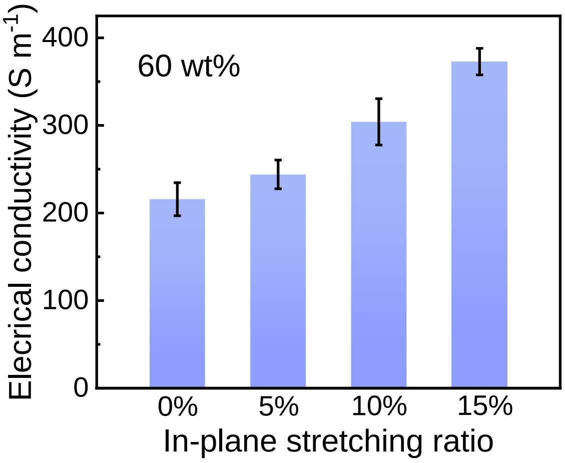

Fig. S9 Electrical conductivity of GNS/ANF-60 wt% films by different in-plane stretching ratio

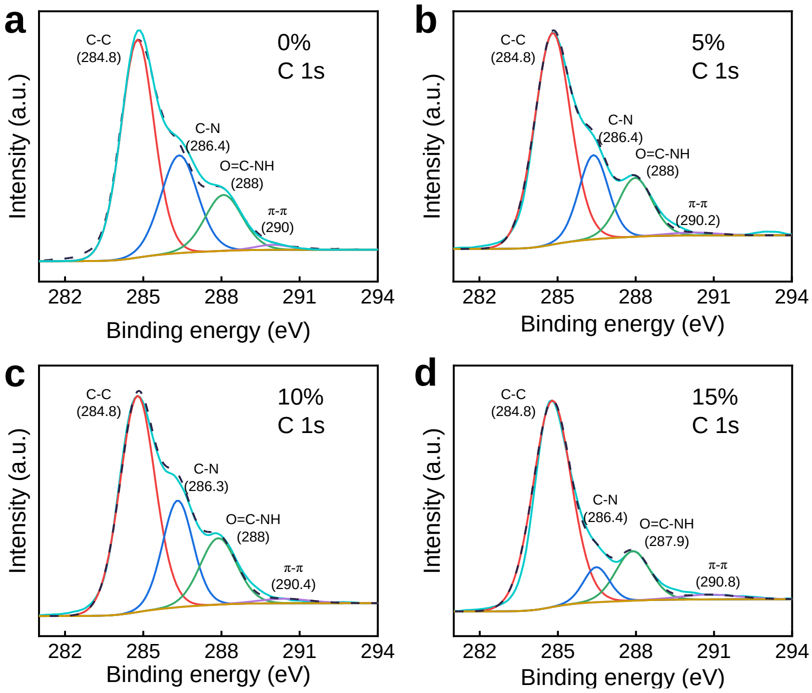

Fig. S10 High-resolution XPS spectra of C 1s for GNS/ANF-60 wt% films by different in-plane stretching ratio. **a** 0%, **b** 5%, **c** 10%, **d** 15%

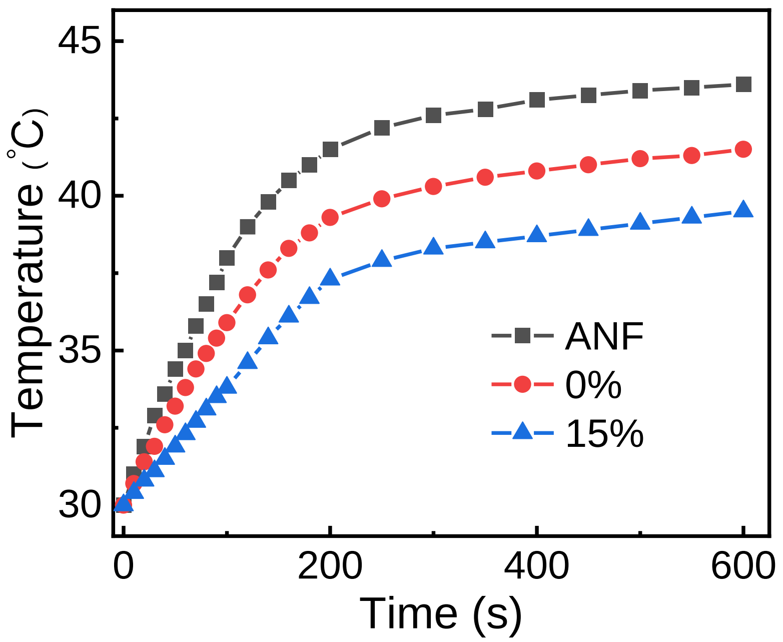

Fig. S11 Temperature evolution curves of smartphones with working time

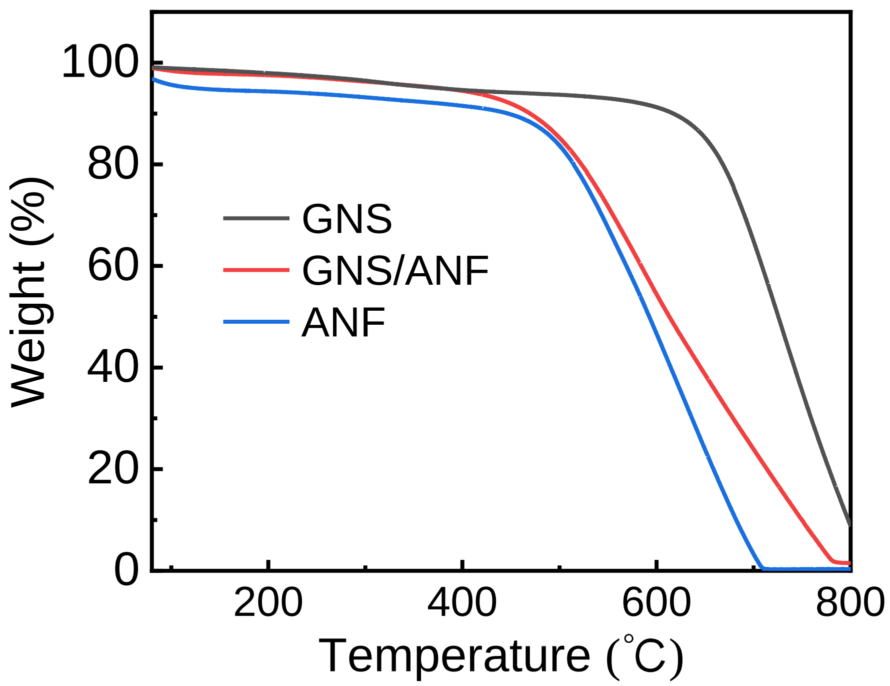

Fig. S12 TGA curves of GNS, GNS/ANF-60 wt% and ANF

Supplementary Tables

Table S1 Fillers, nanosheets conformation, matrix, in-plane thermal conductivity (*K*) and tensile strength (σ) of GNS/ANF composite films with eliminated nanosheets wrinkles were compared with those of thermally conductive composite films with nanosheets wrinkles

Fillers	Nanosheets conformation	Matrix	In-plane *K* (W m^{-1} K^{-1})	σ (MPa)	Refs.
73.3 wt% GF	wrinkles	PVA	21.6	84.4	[S1]
93 wt% GF	wrinkles	PVA	61.3	28	[S1]
40 wt% GF	wrinkles	ANF	48.2	188	[S2]
70 wt% CPGO	wrinkles	CNF	12.8	33.5	[S3]
30 wt% RGO	wrinkles	CNF	6.2	90	[S4]
32.8 wt% GNS	wrinkles	PVA	45.8	177.8	[S5]
55.7 wt% GNS	wrinkles	PVA	71.2	229.4	[S5]
61 wt% GNS	wrinkles	PVA	82.4	259	[S5]
10 wt% GNS	wrinkles	PVA	13.8	84	[S6]
50 wt% GNS	wrinkles	PI	65	102	[S7]
80 wt% GNS	wrinkles	ANF	87.1	55	[S8]
50 wt% GNS	wrinkles	ANF	76.9	145.1	[S9]
60 wt% GNS	wrinkles	ANF	85.6	134.5	[S9]
60 wt% GNS	wrinkles	ANF	85.3	266	[S10]
75 wt% GNS	wrinkles	CNF	58	50.7	[S11]
61 wt% GNS	wrinkles	CNF	134.1	51.9	[S12]
75 wt% GNS	wrinkles	CNF	59.5	46.4	[S13]
40 wt% GNS	wrinkles	PBONF	87.2	188.9	[S14]
50 wt% GNS	wrinkles	PBONF	98.7	194.7	[S14]
30 wt% MXene@Ag	wrinkles	CNF	22.4	71.4	[S15]
60 wt% MXene	wrinkles	CNF	14.9	114.4	[S16]
**60 wt% GNS**	**Eliminated wrinkles**	**ANF**	**146**	**207**	**This work**

GF: Fluorinated graphene; CPGO: Casein phosphopeptide-biofunctionalized graphene oxide nanoplatelets; RGO: Reduced graphene oxide; GNS: Graphene nanosheets; PVA: Poly(vinyl alcohol); PI: Polyimide; ANF: Aramid nanofiber; CNF: Cellulose nanofiber; PBONF: poly(p-phenylene benzobisoxazole) nanofiber

Table S2 Thermal conductivity of constrained dried GNS/ANF-60 wt% films by different in-plane stretching ratio at 25 °C

In-plane stretching ratio	Thermal conductivity (W m^{-1} K^{-1})	
	In-plane	Out-of-plane
0%	81.0 ± 3.0	0.41 ± 0.02
5%	93.9 ± 3.4	0.46 ± 0.02
10%	125.1 ± 3.1	0.58 ± 0.03
15%	146.0 ± 3.5	0.79 ± 0.05

Table S3 Strength at break (σ), Young's modulus (E), strain at break (ε) and toughness (U) of GNS/ANF-60 wt% films by different in-plane stretching ratio

in-plane stretching ratio	σ (MPa)	E (GPa)	ε (%)	U (MJ m^{-3})
0%	99.4 ± 5.5	2.3 ± 0.3	15.4± 0.9	11.2 ± 0.3
5%	122.4 ± 5.6	3.5 ± 0.2	12.6 ± 0.7	10.8 ± 0.6
10%	174.5 ± 5.9	5.5 ± 0.2	9.1 ± 0.5	9.2 ± 0.3
15%	207.0 ± 4.7	8.2 ± 0.5	6.3 ± 0.3	9.1 ± 0.6

Table S4 Assembly, fillers, matrix, in-plane thermal conductivity (K) and tensile strength (σ) of GNS/ANF composite films prepared by in-plane stretching constrained dried strategy were compared with those of thermally conductive composite films prepared by spontaneous dried strategies such as vacuum-assisted filtration, sol-gel-film conversion, layer-by-layer and casting

Assembly	Fillers	Matrix	In-plane K (W m^{-1} K^{-1})	σ (MPa)	Refs.
VAF	93 wt% GF	PVA	61.3	28	[S1]
VAF	80 wt% GNS	ANF	87.2	55	[S8]
VAF	75 wt% GNS	CNF	58	50.7	[S11]
VAF	75 wt% GNS	CNF	59.5	46.4	[S13]
VAF	83 wt% BNNS	CNF	67.6	13	[S17]
VAF	30 wt% BNNS	ANF	46.7	167	[S18]
VAF	50 wt% BNNS	ANF	19.1	59.3	[S19]
VAF	70 wt% BNNS	CNF	30.3	66	[S20]
VAF	60 wt% BNNS	CNF	24.3	45.5	[S21]
Sol-Gel-Film Conversion	40 wt% GF	ANF	20.5	188	[S2]
Sol-Gel-Film Conversion	60 wt% GNS	ANF	85.6	134.5	[S9]
Sol-Gel-Film Conversion	50 wt% BNNS	ANF	64.1	65.6	[S22]
Sol-Gel-Film Conversion	10 wt% BNNS	PBONF	21.3	206	[S23]
Layer-by-Layer	55.7 wt% GNS	PVA	71.2	229.4	[S5]
Layer-by-Layer	32.8 wt% GNS	PVA	45.8	177.8	[S5]
Casting	50 wt% GNS	PI	65	102	[S7]
**In-plane stretching**	**60 wt% GNS**	**ANF**	**93.9**	**122.4**	**This work[1]**
**In-plane stretching**	**60 wt% GNS**	**ANF**	**125.1**	**174.5**	**This work[2]**
**In-plane stretching**	**60 wt% GNS**	**ANF**	**146**	**207**	**This work[3]**

VAF: Vacuum-assisted filtration; This work1, this work2 and this work3: Constrained dried GNS/ANF-60 wt% films by in-plane stretching for (1) 5%, (2) 10%, and (3) 15%.

Supplementary References

[S1] X. Wang, P. Wu, Highly thermally conductive fluorinated graphene films with superior electrical insulation and mechanical flexibility. ACS Appl. Mater. Interfaces **11**(24), 21946-21954 (2019). https://doi.org/10.1021/acsami.9b07377

[S2] M. C. Vu, D. Mani, T.-H. Jeong, J.-B. Kim, C.-S. Lim et al., Nacre-inspired nanocomposite papers of graphene fluoride integrated 3d aramid nanofibers towards heat-dissipating applications. Chem. Eng. J. **429**, 132182 (2022). https://doi.org/10.1016/j.cej.2021.132182

[S3] Y. Liu, M. Lu, Z. Hu, L. Liang, J. Shi et al., Casein phosphopeptide-biofunctionalized graphene oxide nanoplatelets based cellulose green nanocomposites with simultaneous high thermal conductivity and excellent flame retardancy. Chem. Eng. J. **382**, 122733 (2020). https://doi.org/10.1016/j.cej.2019.122733

[S4] N. Song, D. Jiao, P. Ding, S. Cui, S. Tang et al., Anisotropic thermally conductive flexible films based on nanofibrillated cellulose and aligned graphene nanosheets. J. Mater. Chem. C **4**(2), 305-314 (2016). https://doi.org/10.1039/c5tc02194d

[S5] Q. Chen, Z. Ma, Z. Wang, L. Liu, M. Zhu et al., Scalable, robust, low‐cost, and highly thermally conductive anisotropic nanocomposite films for safe and efficient thermal management. Adv. Funct. Mater. **32**(8), 2110782 (2022). https://doi.org/10.1002/adfm.202110782

[S6] Y. Zhuang, K. Zheng, X. Cao, Q. Fan, G. Ye et al., Flexible graphene nanocomposites with simultaneous highly anisotropic thermal and electrical conductivities prepared by engineered graphene with flat morphology. ACS Nano **14**(9), 11733-11742 (2020). https://doi.org/10.1021/acsnano.0c04456

[S7] Q. Wei, S. Pei, X. Qian, H. Liu, Z. Liu et al., Superhigh electromagnetic interference shielding of ultrathin aligned pristine graphene nanosheets film. Adv. Mater. **32**(14), 1907411 (2020). https://doi.org/10.1002/adma.201907411

[S8] K. Xie, Y. Liu, Y. Tian, X. Wu, L. Wu et al., Improving the flexibility of graphene nanosheets films by using aramid nanofiber framework. Compos. Part A Appl. Sci. Manuf. **142**, 106265 (2021). https://doi.org/10.1016/j.compositesa.2020.106265

[S9] L. Huang, G. Xiao, Y. Wang, H. Li, Y. Zhou et al., Self-exfoliation of flake graphite for bioinspired compositing with aramid nanofiber toward integration of mechanical and thermoconductive properties. Nano-Micro Lett. **14**(1), 168 (2022). https://doi.org/10.1007/s40820-022-00919-0

[S10] M. C. Vu, P. J. Park, S.-R. Bae, S. Y. Kim, Y.-M. Kang et al., Scalable ultrarobust thermoconductive nonflammable bioinspired papers of graphene nanoplatelet crosslinked aramid nanofibers for thermal management and electromagnetic shielding. J. Mater. Chem. A **9**(13), 8527-8540 (2021). https://doi.org/10.1039/d0ta12306d

[S11] F. Wang, L. T. Drzal, Y. Qin, Z. Huang, Multifunctional graphene nanoplatelets/cellulose nanocrystals composite paper. Compos. B Eng. **79**, 521-529 (2015). https://doi.org/10.1016/j.compositesb.2015.04.031

[S12] X. Zhang, J. Li, Q. Gao, Z. Wang, N. Ye et al., Nerve-fiber-inspired construction of 3d graphene "tracks" supported by wood fibers for multifunctional biocomposite with metal-level thermal conductivity. Adv. Funct. Mater. **33**(18), 2213274 (2023). https://doi.org/10.1002/adfm.202213274

[S13] G. Li, X. Tian, X. Xu, C. Zhou, J. Wu et al., Fabrication of robust and highly thermally conductive nanofibrillated cellulose/graphite nanoplatelets composite papers. Compos. Sci. Technol. **138**, 179-185 (2017). https://doi.org/10.1016/j.compscitech.2016.12.001

[S14] Y. Wang, S. Xia, H. Li, J. Wang, Unprecedentedly tough, folding-endurance, and multifunctional graphene‐based artificial nacre with predesigned 3d nanofiber network as matrix. Adv. Funct. Mater. **29**(38), 1903876 (2019). https://doi.org/10.1002/adfm.201903876

[S15] E. Jiao, K. Wu, Y. Liu, M. Lu, H. Zhang et al., Robust bioinspired mxene-based flexible films with excellent thermal conductivity and photothermal properties. Compos. Part A Appl. Sci. Manuf. **143**, 106290 (2021). https://doi.org/10.1016/j.compositesa.2021.106290

[S16] E. Jiao, K. Wu, Y. Liu, M. Lu, Z. Hu et al., Ultrarobust mxene-based laminated paper with excellent thermal conductivity and flame retardancy. Compos. Part A Appl. Sci. Manuf. **146**, 106417 (2021). https://doi.org/10.1016/j.compositesa.2021.106417

[S17] Q. Yan, W. Dai, J. Gao, X. Tan, L. Lv et al., Ultrahigh-aspect-ratio boron nitride nanosheets leading to superhigh in-plane thermal conductivity of foldable heat spreader. ACS Nano **15**(4), 6489-6498 (2021). https://doi.org/10.1021/acsnano.0c09229

[S18] K. Wu, J. Wang, D. Liu, C. Lei, D. Liu et al., Highly thermoconductive, thermostable, and super-flexible film by engineering 1d rigid rod-like aramid nanofiber/2d boron nitride nanosheets. Adv. Mater. **32**(8), 1906939 (2020). https://doi.org/10.1002/adma.201906939

[S19] L.-H. Zhao, L. Wang, Y.-F. Jin, J.-W. Ren, Z. Wang et al., Simultaneously improved thermal conductivity and mechanical properties of boron nitride nanosheets/aramid nanofiber films by constructing multilayer gradient structure. Compos. B Eng. **229**, 109454 (2022). https://doi.org/10.1016/j.compositesb.2021.109454

[S20] K. Wu, J. Fang, J. Ma, R. Huang, S. Chai et al., Achieving a collapsible, strong, and highly thermally conductive film based on oriented functionalized boron nitride nanosheets and cellulose nanofiber. ACS Appl. Mater. Interfaces **9**(35), 30035-30045 (2017). https://doi.org/10.1021/acsami.7b08214

[S21] K. Wu, P. Liao, R. Du, Q. Zhang, F. Chen et al., Preparation of a thermally conductive biodegradable cellulose nanofiber/hydroxylated boron nitride nanosheet film: The critical role of edge-hydroxylation. J. Mater. Chem. A **6**(25), 11863-11873 (2018). https://doi.org/10.1039/c8ta03642j

[S22] G. Xiao, J. Di, H. Li, J. Wang, Highly thermally conductive, ductile biomimetic boron nitride/aramid nanofiber composite film. Compos. Sci. Technol. **189**, 108021 (2020). https://doi.org/10.1016/j.compscitech.2020.108021

[S23] Y. Chen, H. Zhang, J. Chen, Y. Guo, P. Jiang et al., Thermally conductive but electrically insulating polybenzazole nanofiber/boron nitride nanosheets nanocomposite paper for heat dissipation of 5g base stations and transformers. ACS Nano **16**(9), 14323-14333 (2022). https://doi.org/10.1021/acsnano.2c04534
